# A randomized controlled trial of remote monitoring for cardiac implantable loop recorders: stay-away trial

**DOI:** 10.1007/s10840-026-02375-x

**Published:** 2026-07-17

**Authors:** John L. Fitzgerald, Austin N. May, David Baker, Stuart Sugito, Amardeep Amardeep, Andrew J. Boyle, Aaron L. Sverdlov, Nicholas Collins, Gwilym M. Morris, Nicholas Jackson, Malcolm Barlow, Stuart Turner, James W. Leitch, Bradley Wilsmore

**Affiliations:** 1 Cardiovascular Department, John Hunter Hospital, Lookout Road, New Lambton Heights, Newcastle, 2305 Australia; 2 University of Adelaide, Port Road, Adelaide, 5000 Australia; 3 School of Medicine and Public Health, Newcastle University, University Drive, Callaghan, Newcastle, 2308 Australia; 4 Hunter Medical Research Institute, Newcastle, Australia

**Keywords:** Implantable loop recorder, Implantable cardiac monitoring, Remote monitoring, Randomized controlled trial, Cost

## Abstract

**Background and aims:**

The benefits of remote monitoring (RM) for pacemakers and defibrillators have been established. It has not been shown that these benefits apply to implantable loop recorders (ILRs). We aimed to investigate the effect of RM on patient outcomes, time spent, and cost during follow-up of ILRs.

**Methods:**

We conducted a multi-centre, randomized, controlled, open-label trial of RM versus office-based follow-up for ILRs. At implant, patients were randomized to RM only, or office visits at 6 weeks, 6 months, and 12 months (no-RM). Primary efficacy endpoints were: (1) time from event to diagnosis, (2) number of related hospital presentations. Secondary endpoints were time spent on follow-up, satisfaction, and cost.

**Results:**

One hundred consecutively referred patients, aged 61 ± 18yrs, 53% male, were recruited between April 2018 and September 2019 (pre-COVID pandemic). Follow-up was 12 months. Patients with RM versus no-RM had less time from event to diagnosis (RM 0(0–0) days, no-RM 40(33–42) days, *P* < 0.001), fewer arrhythmia-related hospital presentations (RM 0.7 ± 1.1, no-RM 2.8 ± 2.6, *P* < 0.019), and lower (better) satisfaction survey scores (RM 13 ± 4, no-RM 16 ± 5; *P* = 0.041). Follow-up clinician time spent (RM 33 ± 29 min, no-RM 29 ± 26 min, *P* = 0.063) and cost of follow up (RM $803, no-RM $1,090; *P* = 0.347) were not different.

**Conclusions:**

In patients with ILRs randomized to receive RM, there was less time from event to diagnosis, there were fewer hospital and clinic presentations, and improved patient satisfaction. RM did not take more time and was cost effective in the Australian health care system. This study provides support for RM of ILRs.

**Registration:**

ACTRN12618001359224.

**Graphical Abstract:**

Overview of this randomized trial with 100 consecutive patients enrolled, randomized 1:1 to remote monitoring only (exception-based care) or office-based follow-up

**Supplementary Information:**

The online version contains supplementary material available at https://doi.org/10.1007/s10840-026-02375-x.

**Figure:**
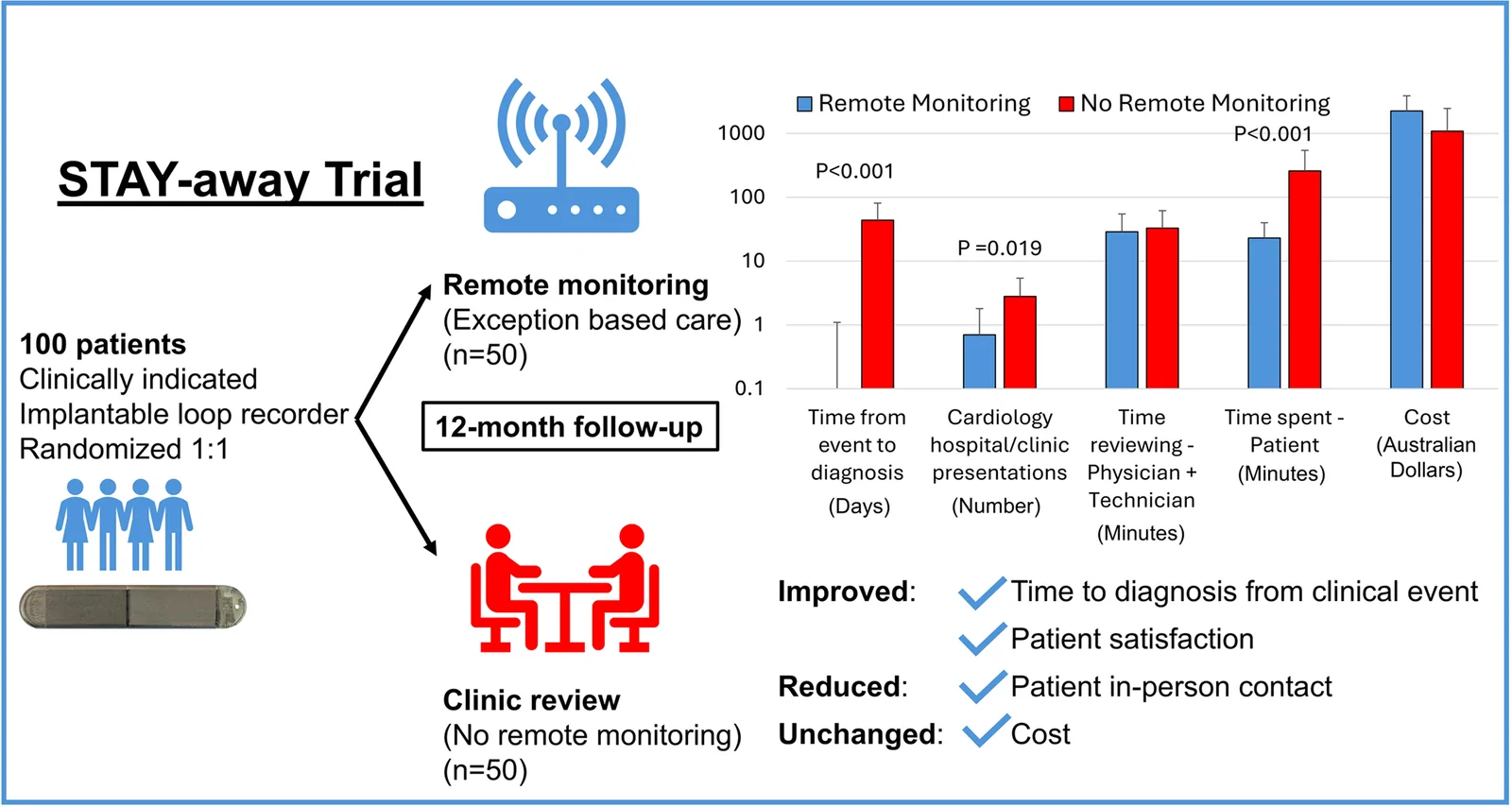

## Introduction

Implantable loop recorders (ILR) are used to detect arrhythmias where symptoms are too infrequent to be captured on non­implantable cardiac rhythm monitoring devices [1]. Clinical studies have evaluated ILRs in the context of syncope [2], recurrent palpitations [3] and AF detection after stroke [4]. Remote monitoring of other cardiac implantable electronic devices (pacemakers and defibrillators) has become the standard of care, based on several randomized trials showing benefits extending across morbidity, mortality, cost effectiveness, and patient satisfaction [5–10]. Remote monitoring for ILRs may differ to pacemakers and defibrillators due to the potential for false-positive detection (over- and under-sensing), symptom activation, and differences in patient populations receiving cardiac monitors compared with pacemakers/defibrillators. Although current consensus is: ‘Implantable loop recorders require immediate connectivity to RM with special programming needs based on the patient’s clinical indication for the implantable loop recorder’ [6] with a class 1 (highest) recommendation, this has the lowest level of evidence ‘EO’ (expert opinion) [11] and the benefits of remote monitoring of ILRs have not been clearly established. We aimed to assess the efficacy, time, patient-satisfaction, and cost of a remote monitoring strategy for ILRs, compared with standard follow-up.

## Methods

We performed a multi-centre, randomized, open-label, study assessing the efficacy, time to diagnosis, patient-satisfaction, and cost of remote monitoring follow-up of ILRs compared to usual office-visit follow-up. Consecutive patients were screened at insertion of ILR for standard indications (syncope or embolic stroke of undetermined source) across two separate sites. Inclusion criteria were age 17–100 years, and the ability to provide informed consent. Exclusion criteria were current pregnancy or breast-feeding and the presence of intellectual or mental impairment. Consenting participants were randomized 1:1 to have active remote monitoring of the ILR with a supplied remote monitoring device and a single phone call follow-up at 6 weeks (Exception based care - no scheduled office visits, ongoing remote monitoring: RM), versus office visits only (no remote monitoring: no-RM) for review of symptomatic events and routinely at 6 weeks, 6 months, and 12 months.

### Endpoints

Primary endpoints included: (1) time from event to diagnosis, where events included both asymptomatic and symptomatic arrhythmias requiring intervention, (2) arrhythmia-related total hospital presentations. Secondary endpoints were: (1) patient time spent on follow-up, (2) patient satisfaction (measured by custom-designed Likert-scale based surveys, see supplement), (3) clinician time spent on follow-up (in person review and time spent performing/reviewing remote monitoring). For patient time, the time spent on arrhythmia-related unscheduled emergency presentations and admissions was not included. For clinician time, travel time, and administration (sending letters, and scheduling appointments) were not included, and (4) Cost. Total cost included: scheduled in-office visits, unscheduled arrhythmia-related in-office visits, arrhythmia-related ED presentations and hospital admissions, remote monitoring fee, remote monitoring transmitter fee. The cost associated with cardiovascular events (AF, stroke, medication changes, adverse effects of medication changes, pacemakers, defibrillators, and ablation) was not included. Costs were calculated using a fee of $115.90 for an in-office visit (MBS items 116 and 11731, ) [12], Transmitter device cost $1450 (private prosthesis listing price) [13], cost of an emergency visit for syncope $991 (AECC classification, 1 July 2021, Item F73b, ARDRV V10.0) and cost of an admission for syncope $1,713 (AR-DRG).

All patients gave written informed consent, and all patient information was anonymised. The study was approved by the Hunter New England Human Research Ethics Committee for both institutions involved (John Hunter Hospital and Lingard Private Hospital) and complied with the Declaration of Helsinki.

## Power calculation and statistics

A sample size of 100 participants was calculated to provide > 80% power to detect a treatment difference of at least 10% reduction in the primary efficacy endpoint (time from event to diagnosis), assuming a time from event to diagnosis of 30 days (± 7) [8, 14] in the no-RM group. Power was calculated for a two-sided t-test with a 5% Type I error rate.

Continuous variables are reported as mean ± standard deviation or as median and interquartile range as appropriate. Normally distributed variables are compared using the unpaired Student’s t-test, with comparisons between both the groups otherwise performed using the Mann–Whitney U test. Categorical variables are stated as absolute and relative frequencies and compared using the χ2 test. All tests are two-tailed. A P-value of < 0.05 is considered statistically significant. Statistical analysis was performed using IBM SPSS version 27 (IBM Corp, 2020, Armonk, NY).

## Results

**Fig. 1:**
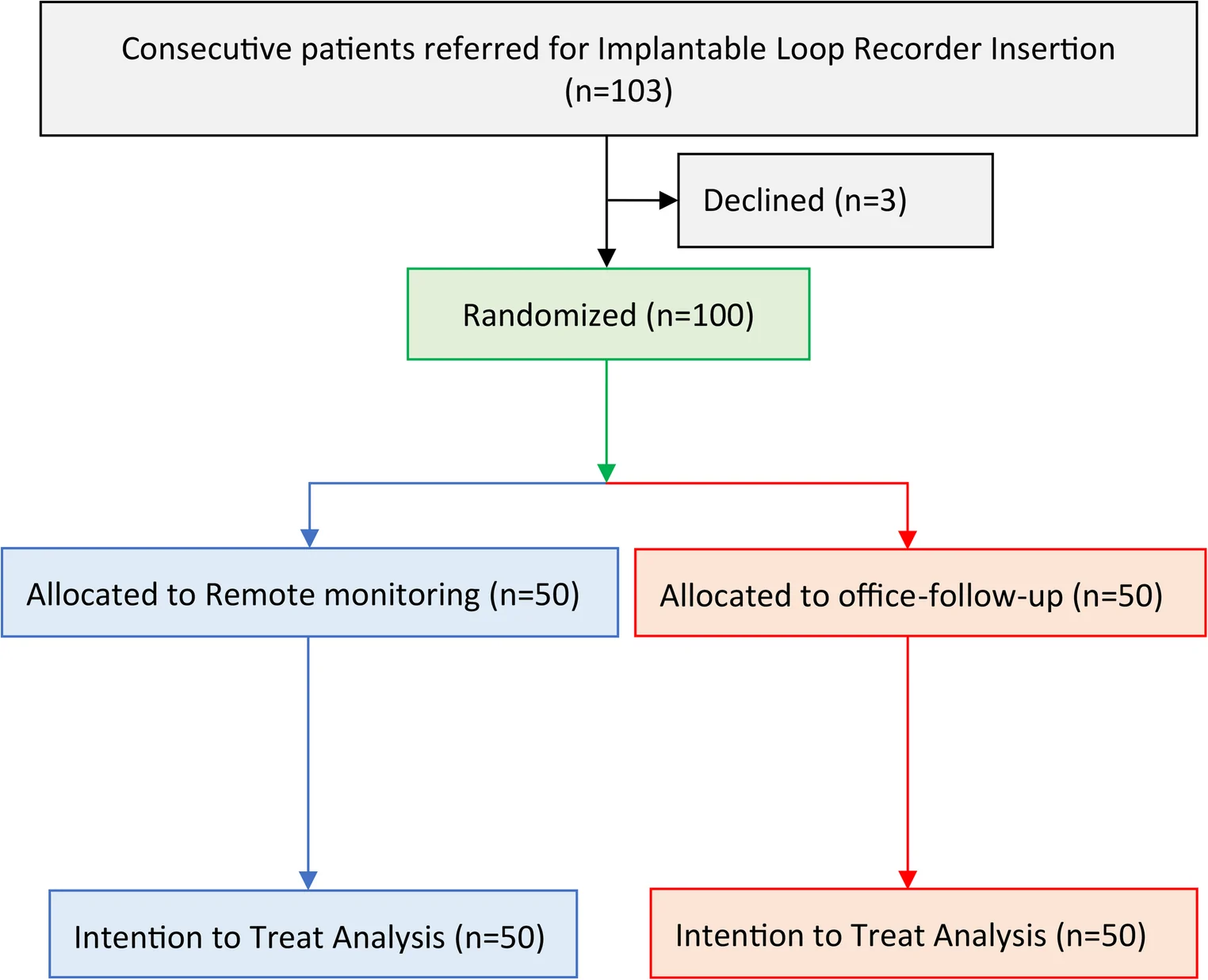
Consort diagram. A total of 103 eligible patients were screened, with 100 enrolled after 3 patients declined participation. Randomization 1:1 with 50 participants allocated to remote monitoring (exception-based care) and 50 participants to office-follow-up with no remote monitoring

One hundred participants across two institutions (85% site 1, 15% site 2), were recruited between April 2018 and September 2019, with follow-up concluded in February 2020. The mean age of participants was 61 ± 17yrs, and 53% were male. The indication for ILR was syncope in 80 participants and embolic stroke of undetermined source in the other 20. There were more men in the no-RM group, otherwise there were no significant between-group differences in baseline characteristics (Table 1). Three patients declined enrolment during the recruitment period. The mean duration of study follow-up was 12 ± 5 months. No patients crossed over between groups and 100% patient follow-up was achieved. There were no serious adverse events related to ILR implant. Minor adverse events included antibiotic requirement in three participants for superficial, ‘possible’ skin infection (none requiring device removal); device-site discomfort beyond 7 days post-implant requiring review in 2 participants, but no device removal was necessary. A consort diagram of patient recruitment is shown in Fig. 1, and trial summary in Graphical Abstract.

**Table 1:** Baseline characteristics*

	Remote monitoring	No remote monitoring	
	*n* = 50	*n* = 50	*P*-value
Age, years	61.8 ± 17.6	60.0 ± 17.6	0.598
Male, n, (%)	21 (42)	32 (64)	0.028
BMI	29.0 ± 5.9	29.0 ± 5.7	0.971
LVEF	60%±7%	60%±9%	0.867
CHA_2_DS_2_-VASc Score	2.7 ± 1.7	2.8 ± 1.7	0.766
ILR Indication: Syncope, n, (%)	40 (80)	40 (80)	1.000
ILR Indication: ESUS, n, (%)	10 (20)	10 (20)	1.000
Antiplatelets, n, (%)	23 (46)	25 (50)	0.689
Anticoagulation, n, (%)	8 (16)	5 (10)	0.372
Rate control, n, (%)	18 (36)	18 (36)	1.000
Smoking, n, (%)	2 (4)	8 (16)	0.169
T2DM, n, (%)	9 (18)	10 (20)	0.650
AF, n, (%)	4 (8)	1 (2)	0.053
Systolic Blood pressure (Mean)	130 ± 19	138 ± 19	0.570
IHD, n, (%)	8 (16)	12 (24)	0.317

* Plus-minus values are mean ± SD

*AF* atrial fibrillation, *BMI* body mass index (kg/m^2^), *ESUS* embolic stroke of undetermined source, *IHD* ischemic heart disease, *ILR* implantable loop recorder, *LVEF* left ventricular ejection fraction, *T2DM* type 2 diabetes mellitus

## Diagnoses on ILR

Patients with remote monitoring (RM) compared to no remote monitoring (no-RM) had a shorter time from event to diagnosis (RM 0 (0–0) days, no-RM 40 (33–42) days, *P* < 0.001, (Table 2). The number of diagnoses made (arrhythmia or not) did not differ between groups (Table 2). There were 7 significant arrhythmia diagnoses in the RM group and 5 in the no-RM group (*P* = 0.538). Time to significant arrhythmia diagnosis was 84 ± 83 days (RM) and 53 ± 32 days (no-RM; *P* = 0.441). The number of patients with confirmation of significant symptoms without rhythm disturbance was 7 (RM) and 6 (no-RM), and the time to this occurring was 181 ± 108 days (RM) and 112 ± 93 days (no-RM, *P* = 0.266).

**Table 2:** Outcomes*

Outcome	Follow-up strategy		*P*-value
	Remote monitoring	No remote monitoring	
*Primary*			
Time from event to diagnosis	0 (0–0) days	40 (33–42) days	*P* < 0.001
Arrhythmia-related hospital presentations	0.7 ± 1.1	2.8 ± 2.6	*P* = 0.019
*Secondary*			
Scheduled in-office visit	0.00 ± 0.00	1.94 ± 1.46	*P* < 0.001
Unscheduled in-office visits	0.06 ± 0.24	0.10 ± 0.30	*P* = 0.143
Emergency Presentations – related	0.52 ± 0.79	0.62 ± 1.1	*P* = 0.426
Admissions (related)	0.16 ± 0.62	0.14 ± 0.41	*P* = 0.643
Overall diagnostic yield^a^	28%	22%	*P* = 0.493
Patient satisfaction^b^ (lower = more satisfied)	13 ± 4	16 ± 5	*P* = 0.041
Remote Monitoring Transmissions	5.5 (2–47)	n/a	n/a
Set-up (no company involvement)	20 ± 16 min (range 5–60)	n/a	n/a
Time to Pacemaker implant if indicated			
Time from implant-device detection	35 ± 3 days	19 ± 6 days	*P* = 0.12
Time from device detection-pacemaker	8 (6–48) days	54 (28–81) days	*P* = 0.76
Time to non-rhythm diagnosis confirmed			
Time from implant-diagnosis normal rhythm	380 (258–502) days	305 (214–396) days	*P* = 0.83
Time to AF detection			
Time from implant-AF detection	54 (40–115) days	2 (2–9) days	*P* = 0.23
Time AF detection-anticoagulation prescription	0 ± 0 days	38 ± 5 days	*P* = 0.005
Diagnoses/Procedures			
Sinus node dysfunction	2	1	
Complete heart block	0	1	
Vasovagal syncope recurrent long pauses	1	0	
Atrial Fibrillation	3	3	
Pacemaker implanted	3	2	
Non-rhythm-related diagnosis			
Seizure disorder (confirmed)	2	2	
Time			
Time spent – Patient	23 ± 17 min	260 ± 285 min	*P* < 0.001
Time reviewing – Physician + Technician	33 ± 29 min	29 ± 26 min	*P* = 0.063
Cost^c^ - including remote monitor	$2,253 ± 1648	$1,090 ± 1381	*P* < 0.001
Cost^c^ - excluding remote monitor	$803 ± 1648	$1,090 ± 1381	*P* = 0.347

* Values are presented as mean +/- standard deviation or median (quartile 1 – quartile 3) where non-normally distributed

^a^ Includes significant arrhythmia diagnosis and significant symptom episode with no arrhythmia recorded; ^b^ Range 0–20; ^c^ All costs are in Australian Dollars

## Interventions resulting from ILR diagnoses

The number of procedures (pacemakers, ablations) did not differ between groups (Table 2). The rate of pacemaker implantation was 5% overall (6.3% of those implanted for syncope), with all of these occurring in implants for syncope (RM 3, no-RM 2; *P* = 0.646). Six patients were anticoagulated for ILR-detected AF (RM 3, no-RM 3; *P* = 1.00), with 5 of these in implants after embolic stroke of undetermined source (13%).

### Hospital presentations

There were fewer cardiology-related hospital presentations overall in patients with remote monitoring (RM 0.7 ± 1.1, no-RM 2.8 ± 2.6, *P* = 0.019, (Table 2). This result was primarily driven by fewer scheduled in-person device checks. There were no differences in any of the other components (unscheduled in-office visits, arrhythmia-related emergency presentations, total arrhythmia-related admissions for inpatient care). In the no-RM group, 84% (97 of 115) of scheduled follow-up appointments were attended.

## ILR transmissions

For those who had remote monitoring, the median number of transmissions was 5.5 (Interquartile range, IQR 2–47) (RM group only: Range 0-1941. Eight patients required a significant change in management (3 pacemakers, 3 anticoagulation, 2 prolonged consultations (> 20 min) with medication changes). Twenty-one alerts required more than 5 min of time.

## Cost

**Fig. 2:**
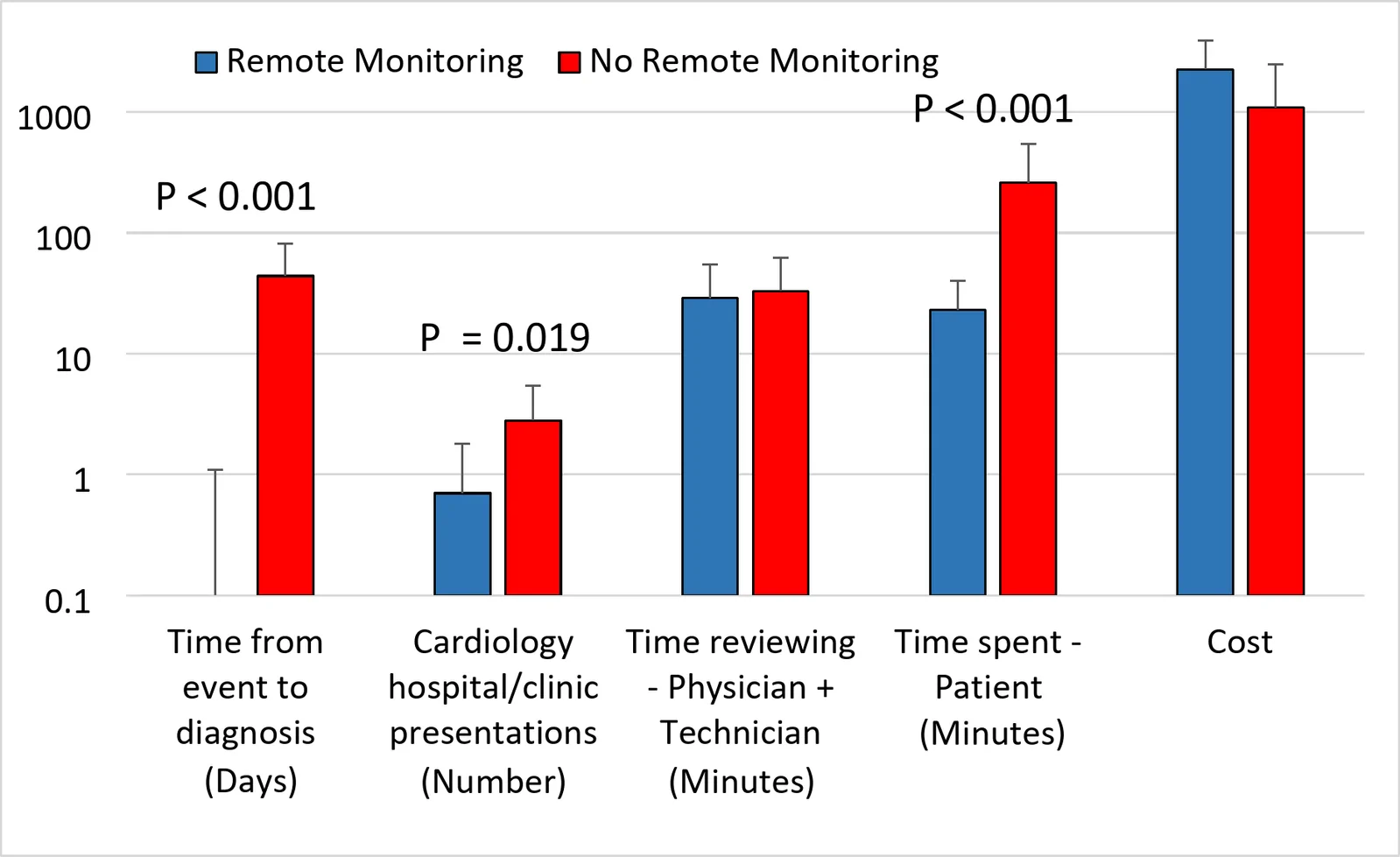
Outcomes of exception-based care (with remote monitoring). Statistically significant (*P* < 0.05) P values shown

The total cost of follow-up was not significantly different between the groups (RM $2,253 ± 1648, no-RM $1,090 ± 1381), however there was marked variability. Cost included the sum of in-person visits ($115.90), arrhythmia-related emergency visits ($991 per visit), arrhythmia-related hospital admissions ($1713), the cost of the remote monitoring transmitter ($1450). It did not include: (a) anticoagulation for earlier detection of AF with remote monitoring, (b) the cost of non-prevented strokes (c) the prevention of syncope with earlier pacemaker and related costs, (d) a cost for performing remote monitoring reviews (not reimbursed at the time of this study in Australia). Figure 2 shows comparisons between groups for the trial outcome measures.

### Patient satisfaction

Participants in the RM group reported higher satisfaction on survey with better (lower) scores compared to participants in the no-RM group (13 ± 4 vs. 16 ± 5 *P* = 0.041) Table 2.

## Discussion

This is the first randomized controlled trial of remote monitoring, without planned in-person review, for implantable loop recorders. It showed improved time from event to diagnosis, and a significant reduction in health care facility visits. Patients with remote monitoring of their ILRs were more satisfied with the service, and this is the first study to show a dramatic reduction in patient time-commitment (4-hour mean difference). Despite the benefits, remote monitoring of ILRs did not cost significantly more, it did not result in additional interventions, and it did not add to the time spent following up patients.

### Differences between monitoring of ILRs and other CIEDs

Whilst remote monitoring of pacemakers and defibrillators is the standard of care based on morbidity, mortality, cost effectiveness, efficiency, and satisfaction, these devices are typically implanted in patients with known cardiac issues, the electrical recordings are intracardiac with different signal-to-noise ratios and with different indications and therefore populations with these devices. Implantable cardiac monitors such as ILRs are electrical loop recorders placed subcutaneously rather than intracardiac, and are more prone to noise, and transmissions can be challenging to diagnose. ILRs are often implanted in younger, healthier patients and in-person reprogramming has reduced clinical benefit relative to pacemakers/defibrillators, therefore evidence for remote monitoring of pacemakers and defibrillations may not apply to ILR. The lack of evidence supporting remote monitoring for ILRs has resulted in less payer support (at the time of this study RM for ILR was not funded in Australia or Europe, unlike USA where it is funded). One propensity score historical cohort-matched study of RM for ILRs has recently shown 2.5 times the chance of evaluation of clinically relevant arrhythmia with RM, but confounding is possible without randomized comparison in a contemporary cohort [15].

### Time to diagnosis and time spent on monitoring

Shorter time from an event to a diagnosis, has been well established for all implanted monitors [8, 14, 16–19]. This study supports those findings, but extends them to show, on average two fewer in-person visits with remote monitoring, without additional presentations to emergency rooms or unplanned in-office visits. Total time for follow- up of ILRs in this study by hospital staff was not different between RM and no-RM as has been shown for pacemakers [20] and defibrillators [21]. However, it is noted that global differences in the involvement of technicians versus physicians varies, and in this study, there was a difference in proportion of time spent by physician and technician, such that if the physician just reviews transmission concerns via a technician, there was no difference in physician time compared with in-person visits (no-RM), whereas if the physician takes on additional burden (manages all remote transmissions, and set- up and phone calls regarding symptoms) then the physician time increases beyond that of in-person checks (with concomitant reduction in technician time). A clinician was involved in reviewing the initial remote monitoring alerts of this particular cohort, whereas those attending clinic were first reviewed by a cardiac technician, then over-read by a clinician. Any query about individual traces was escalated to a senior physician. Patients were contacted (by phone) directly at the time of physician verification of urgent events. Device transmissions were automatic for set criteria, for example pauses > 3 s, atrial fibrillation detection > 30 s within the standard 2-minute sampling window for ILR. Patients could also send symptom transmissions and these were clarified by physician within 24 hours of receipt.

### ILR events leading to pacemaker or defibrillator implantation

In a study with 40 months of follow-up in close to 20,000 patients with ILRs, the rate of pacemaker or implantable cardioverter defibrillator implantation was 21%, with 25% for syncope and 15% for other indications [22]. In comparison, in our study this rate was 5% (6.25% of patients implanted for syncope) over 12 months’ mean follow-up, which is a comparable annualized rate. Meta-analysis for yield from ILRs for syncope including 49 studies in 2017 found a significantly higher proportion of significant arrhythmia diagnosed, at around 45%, with a median time to diagnosis of 145 days, though study heterogeneity was significant and this yield ranged from 12% − 91% [23]. The reasons for these differences in rates may reflect the difference in risk of the underlying populations implanted. For the RM group, the delay from event to detection was 0 days, (see Table 2). Notification was within one business day. Delay to pacemaker implantation, for example for sinus node dysfunction was a feature of severity of symptoms, waiting list times, patients being transported from distant locations, and prioritisation compared to cases with complete AV block waiting as inpatients.

### Signal-to-noise ratio with ILRs

One concern with ILRs is the signal-to-noise ratio with the potential for additional remote alerts. In a recent international study of data from > 26,000 patients monitored via a multi-vendor platform for all CIEDs, ILRs contributed a disproportionately higher rate of transmission data for management compared with other CIEDs [24]. False-positive rates studied recently over 1 month of ILR transmissions were substantial at 46% to 86%, depending on the indication for implant [25]. This highlights the importance of adjudication of transmissions but also the challenge of the significant time required from staff, with increased data to manage. However, our study showed that time was offset by the time taken to see patients in person and perform manual device checks. Artefact was not individually analysed on a per-patient level. On review, 2 patients had significantly higher levels of artefact but due to efficient functioning of the online platform, many of these traces took a few seconds to review. There is also emerging evidence that artificial intelligence-based (AI) algorithms adopted in newer ILRs result in a reduction in the number of false-positives requiring adjudication [26].

### Reduced patient contact due to COVID pandemic

The requirement for remote versus office-based care, has shifted in the recent clinical environment due to the severe respiratory syndrome coronavirus 2 (SARS-CoV-2) global pandemic. With the increased use of telehealth and avoidance of direct person-to-person contact during SARS-CoV-2-related widespread ‘lockdowns’, remote monitoring is likely to have even greater benefit, given this study showed remote monitoring results in fewer in-person, healthcare interactions. Additionally, there is data to suggest a low clinical usefulness with much lower rates of programming change for routine in-person pacemaker checks, compared with remote monitoring-driven checks [16]. This is even more likely to apply to ILRs as outside of detection sensitivity changes to reduce false-positive transmissions, an in-person check provides very little objective benefit compared to remote monitoring. Systematization of approaches to reprogramming appropriately to maintain true event detection but significantly reduce false-positive transmissions is an ongoing challenge but promises to further reduce the time-burden on monitoring services [27]. This study did not involve strategic recall for reprogramming for these reasons. Newer devices introduced since this study incorporate remote programmability along with AI algorithms and have been proven to reduce false-positive transmission data management [28]. This has been shown to reduce the time and cost burden on clinicians and would have likely resulted in a further reduced cost of remote monitoring during this current study, if it had been available at the time [29].

### Implantable loop recorders following embolic stroke

While this study investigated remote monitoring for the two standard reimbursed indications currently (embolic stroke of undetermined source and unexplained recurrent syncope), recent data shows there may be utility to expanding indications in future. The STROKE AF study showed that AF detection in stroke patients with known or presumed atherosclerotic cause for stroke at 12 months was > 12%, and similar to that in cryptogenic stroke in the CRYSTAL AF study which is the seminal study driving the accepted indication for ILR implant in embolic stroke of undetermined source (ESUS) [30]. In the PER DIEM study, with all-comer non-AF stroke (though 66% were ESUS), the 12-month detection rate was even higher at > 15% [31]. The duration of AF necessary to lead to benefit from anticoagulation, however, is not clear, with the recent LOOP, NOAH-AFNET 6 and ARTESIA trials showing no net benefit of anticoagulation for detected AF or atrial high rate episodes > 6 min [32–34]. In our study the time from implant to device detection of AF was actually shorter in the no-RM group, which was influenced by the low event rate, but small number of early detections in the no-RM group. Longer follow-up and/or a larger study would be needed for adequate power to investigate this in more detail. In heart failure patients, a small Dutch study has recently shown remotely monitored ILRs led to a significant proportion of important prognostic arrhythmia detection (including tachycardia, bradycardia/pauses and AF) where none of these were detected on routine physician office visits [35]. The utility of these increased detections in generating treatment changes which actually result in improved outcomes is still an area where data is lacking, and this current study contributes evidence to assess the cost-benefit of the intervention. In this study, the device detection of AF with RM led to immediate anticoagulation initiation. In the No-RM group, although an early AF event occurred and was detected, due to the wait for in-person check, this led to a longer delay to diagnosis confirmation by staff and therefore delay to anticoagulation. A larger sample size would have been required to determine the differences between groups in terms of AF events, and this was beyond the scope of this study.

### Cost considerations

In addition to the time spent on remote monitoring, the cost of the home monitor device is a consideration in many cases. This device may not be supplied with the ILR and may require an additional outlay at the time of implant. While this is a current challenge, the move to smart device, app-based communication with ILRs (and other cardiac devices) may remove the cost of the physical device for many, and reduce electronic waste generated at the end-of-life of these monitors.

Irrespective of the use of RM, there were no differences in arrhythmia detection requiring intervention (e.g., pacemakers, ablations, and anticoagulation requirements) - these were simply identified sooner with remote monitoring. Therefore, whilst the cost of various services differs between countries and health care systems, given the reduction in health care interactions, without increased interventions between the groups, this cost analysis is likely to apply to most health care systems.

### Limitations

This study was open-label, so a bias from remote monitoring cannot be excluded. We tested the difference in strategies rather than the effect only of the remote monitoring data, so this effect is not a confounder, but forms an important part of the results of the study. The in-office follow-up schedule was chosen to reflect local practice and may differ from routine frequency in other centres, potentially affecting generalisability of results. Given the variability in cost, this study was underpowered to detect a difference. However, the cost of the device is likely to be largely irrelevant soon with application based, blue-tooth, smart-phone enabled transmission. The cost analysis was limited to the average rebated cost of an admission and is not individualized. It also only specifically relates to Australian healthcare provision, but with definite global implications. Additionally, this study was not powered to detect the cost of later diagnosis without remote monitoring, and the potential consequences (e.g., stroke from undetected AF). Finally, the time taken to set up an infrastructure for remote monitoring, as well as the time spent by administrative staff sending letters and scheduling appointments, was not included in the time or cost analysis. It is acknowledged that models of remote monitoring and which specific staff are involved vary considerably from centre to centre which will affect the generalisability of these results.

## Conclusions

In this randomized, open-label trial of exception-based care (no planned in-person follow up), with remote monitoring compared with in-person follow up (no remote monitoring) of implantable loop recorders, remote monitoring (exception based care) showed improved time to detection from clinically relevant events, reduced healthcare/in-person interactions, it was not more costly, did not increase the time spent to follow patients with these devices, and importantly, from a patient-centric perspective, it saved significant time for the patient, and increased patient satisfaction.

### Capsule summary

- This is the first randomized controlled trial to investigate remote monitoring vs. office-based care for implantable loop recorders.
- Remote monitoring for implantable loop recorders in this study reduced time from clinical event to diagnosis, reduced in-person healthcare contact and improved patient satisfaction, without increasing cost compared to in-office only follow-up.

## Supplementary Information

Below is the link to the electronic supplementary material.

Supplementary Material 1

## Data Availability

Data are available upon reasonable request to the corresponding author.

## Abbreviations

AF: Atrial fibrillation
CIED: Cardiac Implantable Electronic Device
ILR: Implantable Loop Recorder
No-RM: No remote monitoring
RM: Remote monitoring
