# Supplementary material for: A randomized controlled trial of remote monitoring for cardiac implantable loop recorders: stay-away trial
Source: J Interv Card Electrophysiol. 2026 Jul 17;69(9):2921–9. doi: 10.1007/s10840-026-02375-x (PMC13769693; doi:10.1007/s10840-026-02375-x)
Supplement: Supplementary file 1 [file 10840_2026_2375_MOESM1_ESM.docx]

Supplemental Appendix: Patient Satisfaction Survey – ILR Follow-up strategy pg 1-2

Patient Satisfaction Survey - Implantable Loop Recorder Follow-up Strategy

Patient Satisfaction Survey

Top of Form

1. Overall, how satisfied or dissatisfied are you with this follow-up strategy?

1	Very satisfied

2	Somewhat satisfied

3	Neither satisfied nor dissatisfied

4	Somewhat dissatisfied

5	Very dissatisfied

2. Which of the following words would you use to describe the mode of follow-up?

1	Useful

2	Somewhat useful

3	Neutral

4	Not very useful

5	Not at all useful

3. How well does the follow-up meet your needs?

1	Extremely well

2	Very well

3	Somewhat well

4	Not so well

5	Not at all well

4. How would you rate the efficiency of the follow-up method?

1	Very efficient

2	Efficient

3	Neutral

4	Inefficient

5	Very inefficient

5. How would you rate the user-friendliness of the follow-up method?

1	Excellent

2	Above average

3	Average

4	Below average

5	Poor

6. How easy was it to trouble-shoot issues arising during follow-up?

1	Very easy

2	Somewhat easy

3	Neutral

4	Somewhat difficult

5	Very difficult

7. How would you rate the impact on your health of the follow-up strategy?

1	Very positive impact

2	Positive impact

3	No impact

4	Negative impact

5	Very negative impact

8. How likely are you to continue the follow-up strategy in future, given a choice of strategies?

1	Extremely likely

2	Very likely

3	Somewhat likely

4	Not so likely

5	Not at all likely

9. How likely is it that you would recommend this follow-up strategy to another person with an implantable loop recorder?

1	Very likely

2	Somewhat likely

3	Neither likely nor unlikely

4	Unlikely

5	Very unlikely

10. Do you have any other comments, questions, or concerns?

Bottom of Form
